# Fructose and salt induce sex- and ovary dependent cardiac hypertrophy in Dahl salt-sensitive rats

**DOI:** 10.3389/fcvm.2026.1753554

**Published:** 2026-03-18

**Authors:** M. A. Akhtar, S. Ludvigsen, C. Mancusi, G. de Simone, A. D. Hafstad, E. Gerdts, K. Ytrehus

**Affiliations:** 1 Department of Medical Biology, UiT-The Arctic University of Norway, Tromsø, Norway; 2 Department of Advanced Biomedical Science, University of Naples Frederico II, Naples, Italy; 3 Department of Clinical Science, University of Bergen, Bergen, Norway

**Keywords:** cardiac remodeling, dahl salt-sensitive rats, dietary factors, echocardiography, fructose, gene expression, salt-sensitive hypertension, sex hormones

## Abstract

The consumption of sugar and salt plays a pivotal role in the development of metabolic disorders, hypertension, and subsequent cardiovascular morbidity. This study investigates the influence of sex and gonadal status on early signs of cardiac remodeling in hypertensive-prone Dahl salt-sensitive (DSS) rats by comparing male, female, and ovariectomized (OVX) female rats. DSS rats were provided with 10% fructose in their drinking water and subjected to either a high-salt diet (6% NaCl) or a standard-salt diet (0.3% NaCl) for 8 weeks. Mean arterial pressure, initially 115 ± 2 mmHg at baseline across all groups, increased to 127 ± 5 mmHg with the standard-salt diet and to 156 ± 7 mmHg with the high-salt diet (*p* < 0.001). High-salt intake was associated with significant concentric cardiac remodeling in OVX females and males but not in intact females. In parallel with these cardiac changes, gene expression analysis of left ventricular apex samples revealed increased mRNA levels of natriuretic peptides and genes associated with fibrosis and inflammation. These findings highlight a complex interplay between diet, sex hormones, and the pathophysiology of hypertensive cardiac disease. Further, this study underscores the importance of including both sexes and to consider gonadal status in hypertensive cardiac remodeling.

## 1 Introduction

Cardiovascular disease remains a leading cause of morbidity and mortality worldwide, with hypertension identified as a major risk factor whose prevalence continues to rise globally (1). Hypertension induces structural, functional, and molecular changes in the heart that often lead to heart failure. Salt-sensitive hypertension is a particularly prevalent condition attributed to the complex interplay between dietary factors, genetic predisposition and gonadal status (2). Heart failure pathophysiology and development in general vary depending on what type of stressors the heart is subjected to. Experimental studies have extensively documented the progression of heart failure due to hypertension, but most of these studies have focused on male pathophysiology (3–7). However, sex differences in the progression of cardiovascular diseases in relation to menopausal status have been reported in women (8). Despite this, the mechanisms underlying these differences remain unclear. In women, presence of essential arterial hypertension increases with age and significantly contributes to the development of heart failure (5, 6). Heart hypertrophy with progression to heart failure is replicated experimentally in Dahl salt-sensitive (DSS) rats which serve as a well-established model for studying salt-induced hypertension in genetically predisposed individuals (9, 10). Our previous experimental work showed that in adult female DSS rats, loss of ovarian function exacerbates cardiac remodeling in response to high-slat diet (11). This remodelling is characterized by increased concentric hypertrophy and increase in perivascular fibrosis, occurring prior to the onset of overt heart failure signs.

Changes in gene expressions provide clinical insights into how stressors influence heart remodeling and contribute to understanding sex differences in hypertensive heart disease development. In a previous short communication, we reported that hearts from hypertensive male DSS rats have significantly higher gene expression levels of angiotensin converting enzyme and other renin angiotensin related genes compared to females with intact ovaries (12, 13). Dietary fructose is a mild metabolic stressor which under experimental conditions elevates blood pressure by mechanisms proposed to be related to cellular sodium handling (14). Dietary fructose is also suspected to contribute to hypertension in humans (15, 16). Fructose is a common additive in the modern food industry and one of the two components of table sugar (sucrose). It is metabolized almost exclusively in the liver, bypassing key regulatory steps and rapidly fueling liver *de novo lipogenesis*. This process leads to increased fat production and consequently contributes to hyperlipidemia (17). Experimental research has shown that DSS rats develop gradual onset hypertension when fed a standard diet supplemented with fructose, and an expedited progression when exposed to a high-salt diet (14, 18, 19). In male DSS rats, high intake of sugars such as sucrose and fructose has been shown to accelerate development of heart hypertrophy, and, when fructose feeding is prolonged, progression towards heart failure (20).

Understanding how early subclinical manifestation of hypertensive heart disease are influenced by sex hormones, is critical for advancing knowledge of salt-sensitive hypertension. Therefore, the present study was designed to examine echocardiographic manifestations of early hypertensive heart disease in relation to changes in gene expression in the left ventricle (LV) of male and female DSS rats. This study includes both females with intact ovaries and those with loss of ovarian function, using a combination of fructose supplementation with either normal salt or elevated salt diet.

## 2 Material and methods

### 2.1 Animals and experimental design

DSS rats were obtained from Charles River Laboratories (Kingston location K90, New York, USA). The study included 30 male and 60 female DSS rats, all housed in a temperature-controlled room with a 12 h light/dark cycle. Animals were kept on a standard chow diet containing 0.3% NaCl until they reached the age of 10 weeks. At 9 weeks of age, thirty female rats underwent OVX (21) and were allowed to recover for 1 week before the start of diet intervention. The study was conducted with two cohorts, each consisting of three groups: male, female and OVX female DSS rats. Both cohorts received 10% fructose in drinking water but differed with respect to diet intervention. One cohort received a standard-salt chow (0.3% NaCl), while the other cohort were fed a high-salt diet with chow supplemented with 6% NaCl (Special Diet Services, UK) for 8 weeks, resulting in the following groups: male (DSSM, *n* = 15), female (DSSF, *n* = 15), ovariectomized female (DSS-OVX, *n* = 15), male-NaCl (DSSM-NaCl, *n* = 15), female-NaCl (DSSF-NaCl, *n* = 15), and female OVX-NaCl (DSS-OVX-NaCl, *n* = 15). The study was approved by the Norwegian animal welfare authorities (approval FOTS ID 6784), and all procedures conformed to the guidelines from Directive 2010/63/EU of the European Parliament on the protection of animals used for scientific purposes.

During the period of diet intervention, body weight and blood pressure were measured weekly. Blood pressure were measured by non-invasive tale cuff technique (Coda™ Standard system, Kent Scientific Corporation, Connecticut, USA). The animals were familiarized with handling prior to the start of the intervention. Animals were placed in a heating chambre at 35 °C for 20 min before the procedure to ensure well perfusion of the tail. During the measurement procedure, animals were restrained and placed on a heating pad at 34 °C. A blood pressure cuff was then positioned at the base of the tail, and the conscious animals were covered with blankets to minimize stress. Each session included four habitational measurements followed by ten normal blood pressure measurements.

### 2.2 Echocardiographic assessments

Echocardiography was performed at baseline and endpoint using the Vevo 2100 imaging platform, equipped with an MX 250 transducer (21 MHZ) (Fujifilm Visualsonics Inc., Toronto, Canada), ECG electrodes and accompanying software. Rats were anesthetized with 3% isoflurane in oxygen in an induction chamber, and anaesthesia was maintained with 1.5% isoflurane delivered via a nose cone. Hair from the chest and abdominal areas was removed with an electric shaver, and then depilatory cream was used. Subsequently, electrode gel was applied to each ECG strip, and the rat's limbs were immobilised using tape. Body temperature was constantly monitored by using a rectal probe and maintained at 37 °C using a heated table plate and an adjustable heating lamp. Two-dimensional guided M-mode images of the LV were obtained from the parasternal short-axis view at the level of the papillary muscles. Post-acquisition analysis was performed with the VevoLab software (Visual Sonics®, FujiFilm, Toronto, CA). The M-mode echocardiographic images were used to evaluate LV posterior and anterior wall thickness in systole and diastole (LVPWs, LVAWs, LVPWd and LVAWd), LV internal diameter in systole and diastole (LVIDs and LVIDd), stroke volume (SV), cardiac output (CO), LV ejection fraction (EF), fractional shortening (FS), and LV end-systolic and end-diastolic volume (LVESV and LVEDV), which were calculated using the formula [7× LVID^3^/(2.4 + LVID)]. LV mass was calculated as (0.8424 × [(LVIDd + LVPWd + LVAWd)^3^—LVIDd^3^]). LV mass was indexed for tibia length at endpoint. Although we did not use the more advanced formula developed by Giovanni et al. (22), we found a highly significant correlation between LV weight at autopsy and LV mass (*p* < 0.001, *r*^2^ = 0.7, *n* = 66), using the standard formula in the VEVOLAB software. Relative wall thickness (RWT) was calculated as twice the LVPWd divided by the LVID in diastole (2xLVPWd/LVIDd) (11). Transmitral flow was assessed from the apical four-chamber view to measure isovolumic relaxation time (IVRT). These measurements were performed on at least three heartbeats from each view and then averaged.

Following endpoint echocardiography measurements, rats were euthanized with an intraperitoneal injection of 100 mg/kg sodium pentobarbital. The heart was rapidly excised, atria and right ventricle removed, and LV weighed, and a biopsy was taken from the apex and stored in RNA-later (Qiagen, Hilden, Germany). Additionally, kidney, lungs, and liver were removed and weighed, and the tibia harvested for length measurements. Total body surface area (BSA) at endpoint was calculated based on body weight (W) as = kW^2/3^ with k = 9.83 (23).

### 2.3 Gene expression

Based on our previous studies (11, 24), mRNA expression of a set of selected target genes associated with heart function, structure, inflammation, and fibrosis were examined (Supplementary Table S1). Tissues from the apex biopsies were homogenized and lysed, and total RNA was extracted according to the RNeasyFibrous Tissue protocol (Qiagen). RNA concentration was measured spectrophotometrically (NanoDrop, Witec, Switzerland). Reverse transcription of RNA was carried out using a High-Capacity cDNA Reverse Transcription Kit (Applied Biosystems, Foster City, CA, USA). The Quantitative real-time PCR (qRT-PCR) was performed in a fast real-time thermal cycler (Roche Light Cycling 96) using SYBR green master mix (Fast Start Essential DNA Green Master mix, Roche). Primers were obtained from Eurogentec (Seraing, Belgium) and Sigma-Aldrich (St Louis, Mo, USA). The relative expression ratio of the target gene was calculated using the 2^−ΔΔCT^ method. The expression of the target genes was normalized to stably expressed reference genes SDHA (Succinate Dehydrogenase Complex Flavoprotein Subunit A) and HPRT (Hypoxanthine-guanine phosphoribosyl transferase) as determined by NormFinder (25).

### 2.4 Statistical analysis

Data analysis was performed by using GraphPad Prism 10.3.1 software. Data presented in tables and figures are group averages ± SEM. Endpoint data were analyzed parametrically using two-way ANOVA for the effect of diet (two diet cohorts), the effect of sex (three sex groups) and interaction between sex and diet. A *p-*value < 0.05 was considered statistically significant for all the parameters. When significance was observed, further testing was performed using Tukey's multiple comparisons test for differences between the sex-groups. Correlations between end-point cardiac echocardiographic parameters and myocardial mRNA expression of genes were done using linear regression.

## 3 Results

### 3.1 Mean arterial pressure (MAP), body and organ weights

**Figure 1:**
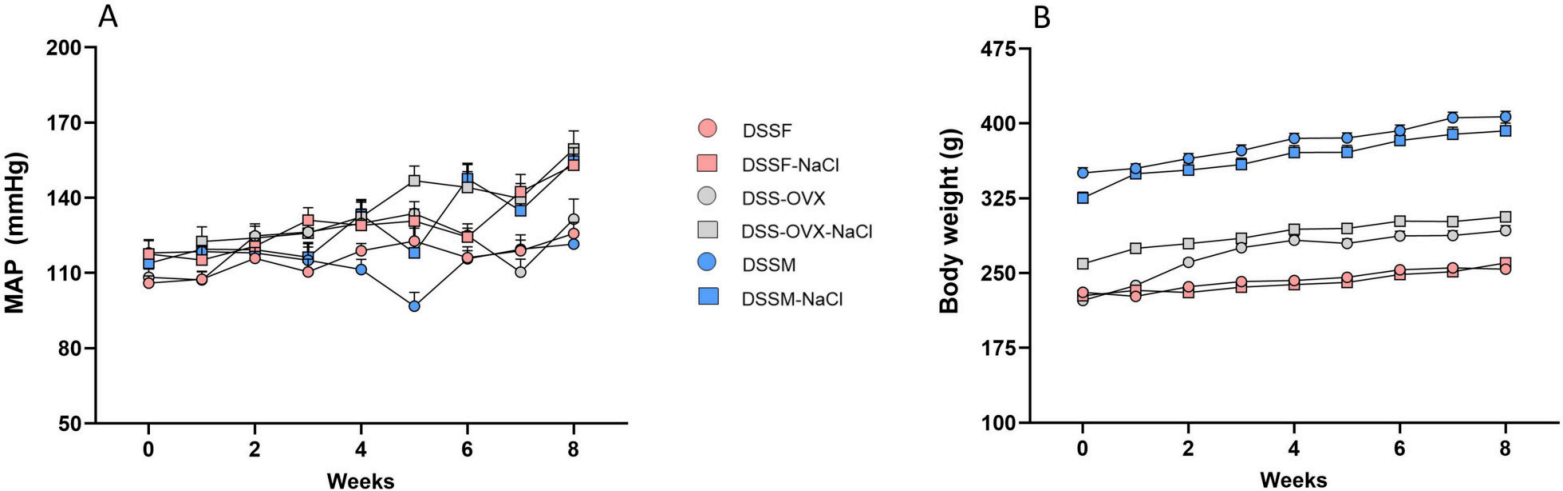
Mean arterial pressure (MAP) **(A)**, and body weight development **(B)**, from Dahl salt-sensitive (DSS) rats following diet intervention (8 weeks). Intact female (DSSF), ovariectomized female (DSS-OVX) and male (DSSM) rats in the control diet group were given standard chow (0.3% NaCl) and water supplemented with 10% fructose, while those in the high salt diet groups received a salt-supplemented diet (6% NaCl) along with fructose supplementation (DSSF-NaCl, DSS-OVX-NaCl and DSSM-NaCl). The values shown mean ± SEM, *n* = 14–15 in each group.

Compared to baseline values, MAP was significantly increased in all groups after 8 weeks of diets, except for DSSM receiving fructose and standard chow (Figure 1A). The high-salt diet significantly increased blood pressure at endpoint across all groups (Table 1).

**Table 1:** End-point measurements of body organ data and blood pressure (BP) obtained from Dahl salt-sensitive (DSS) rats following diet intervention (8 weeks). Intact female (DSSF), ovariectomized female (DSS-OVX) and male (DSSM) rats in the control diet group were given standard chow (0.3% NaCl) and water supplemented with 10% fructose, while those in the high salt diet groups (+NaCl) received a salt-supplemented diet (6% NaCl) along with fructose supplementation. Data are presented as mean ± SEM, *n* = 14–15 in each group.

Parameter	Diet	DSSF	DSS-OVX	DSSM	Effect of diet and/or sex (*p* value)
Systolic BP (mmHg)	Fructose only	156 ± 5	161 ± 8	153 ± 4	Sex n.s.
	Fructose + NaCl	183 ± 7	192 ± 6	186 ± 7	+NaCl *p* < 0.001
Diastolic BP (mmHg)	Fructose only	111 ± 5	117 ± 8	106 ± 4	Sex n.s.
	Fructose + NaCl	138 ± 7	143 ± 8	139 ± 6	+NaCl *p* < 0.001
MAP	Fructose only	126 ± 5	132 ± 8	122 ± 4	Sex n.s.
	Fructose + NaCl	153 ± 7	159 ± 7	144 ± 12	+NaCl *p* < 0.001
Body weight (g)	Fructose only	254 ± 2	293 ± 5*	407 ± 5*^,#^	Sex *p* < 0.001
	Fructose + NaCl	260 ± 4	306 ± 3*	393 ± 7*^,#^	+NaCl n.s.
					Interaction *p* = 0.04
BSA	Fructose only	395 ± 2	433 ± 5*	540 ± 5*^,#^	Sex *p* < 0.001
	Fructose + NaCl	400 ± 4	446 ± 2*	527 ± 7*^,#^	`+NaCl n.s.
					Interaction *p* = 0.01
Left ventricular weight (mg)	Fructose only	759 ± 18	886 ± 22*	1,131 ± 37*^,#^	Sex *p* < 0.001
	Fructose + NaCl	907 ± 25	1,034 ± 25*	1,154 ± 34*^,#^	+NaCl *p* < 0.001
					Interaction *p* = 0.05
LV mass index (mg/cm)	Fructose only	186 ± 4	215 ± 6*	249 ± 9*^,#^	Sex *p* < 0.001
	Fructose + NaCl	220 ± 7	250 ± 6*	255 ± 7*	+NaCl *p* < 0.001
Kidney weight (mg)	Fructose only	990 ± 14	977 ± 16	1,463 ± 22*^,#^	Sex *p* < 0.001
	Fructose + NaCl	1,096 ± 28	1,153 ± 28	1,621 ± 41*^,#^	+NaCl *p* < 0.001
Kidney weight/tibia length (mg/cm)	Fructose only	243 ± 3	238 ± 4	322 ± 6*^,#^	Sex *p* < 0.001
	Fructose + NaCl	266 ± 7	279 ± 7	359 ± 8*^,#^	+NaCl *p* < 0.001
Liver weight (g)	Fructose only	8.8 ± 0.2	10.0 ± 0.2*	16.0 ± 0.4*^,#^	Sex *p* < 0.001
	Fructose + NaCl	10.7 ± 0.3	11.8 ± 0.2*	15.6 ± 0.5*^,#^	+NaCl *p* < 0.001
					Interaction *p* = 0.002
Liver weight/tibia length (g/cm)	Fructose only	2.16 ± 0.04	2.43 ± 0.05*	3.52 ± 0.10*^,#^	Sex *p* < 0.001
	Fructose + NaCl	2.59 ± 0.09	2.85 ± 0.06*	3.44 ± 0.10*^,#^	+NaCl *p* < 0.001
					Interaction *p* = 0.002
Lung weight (mg)	Fructose only	1,843 ± 177	1,707 ± 83	1,905 ± 47	Sex n.s.
	Fructose + NaCl	1,733 ± 120	2,100 ± 161	1,922 ± 36	+NaCl n.s.
Lung weight/tibia length (mg/cm)	Fructose only	451 ± 42	416 ± 21	420 ± 11	Sex n.s.
	Fructose + NaCl	420 ± 30	508 ± 39	426 ± 34	+NaCl n.s.
Tibia length (cm)	Fructose only	4.07 ± 0.02	4.11 ± 0.03	4.54 ± 0.03*^,#^	Sex *p* < 0.001
	Fructose + NaCl	4.13 ± 0.05	4.14 ± 0.03	4.52 ± 0.03*^,#^	+NaCl n.s.

Body surface area (BSA), Mean arterial pressure (MAP). Significant difference *p < 0.05 vs. the DSSF and ^#^*p* < 0.05 vs. the DSS-OVX from the corresponding diet group. Interaction between diet and sex is indicated with *p*-values in the table.

**Figure 2:**
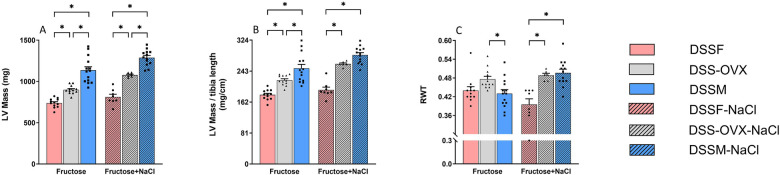
End-point measurements of left ventricular (LV) structural parameters obtained from Dahl salt-sensitive (DSS) rats following diet intervention (8 weeks) using echocardiography from the parasternal short axis view in M-mode among the three sex groups. LV mass **(A)**, LV Mass/tibia length **(B)**, Relative wall thickness **(**RWT, **C)**. Intact female (DSSF), ovariectomized female (DSS-OVX) and male (DSSM) rats in the control diet group were given standard chow (0.3% NaCl) and water supplemented with 10% fructose, while those in the high salt diet groups received a salt-supplemented diet (6% NaCl) along with fructose supplementation (DSSF-NaCl, DSS-OVX-NaCl and DSSM-NaCl). The values are shown as mean ± SEM, *n* = 6–14 in each group. *P < 0.05, 2-way ANOVA with Tukey's *post hoc* test.

Body weight increased significantly in all groups throughout both diets (Figure 1B). There was interaction between the effect of elevated salt and sex at endpoint (Table 1). OVX females developed slightly elevated body weight and liver weight compared to intact females (*p* < 0.05). Males did however exhibit slightly reduced body weight and liver weight in response to high-salt diet. With respect to organ weights at endpoint high-salt diet led to higher LV and kidney weights across groups, but the difference in LV weight was more evident in the two groups of females than in male DSS rats (Table 1). High-salt diet increased liver weight in both female DSS rat groups, but not in male DSS rats (Table 1). Lung weight and tibia length were not influenced by a high-salt diet (Table 1), and lung weights did not differ between groups (Table 1). Female OVX-DSS rats also exhibited higher LV, and liver-weights than female DSS rats with intact ovaries in both diet intervention cohorts (Figure 2 and Table 1).

### 3.2 Echocardiography

**Figure 3:**
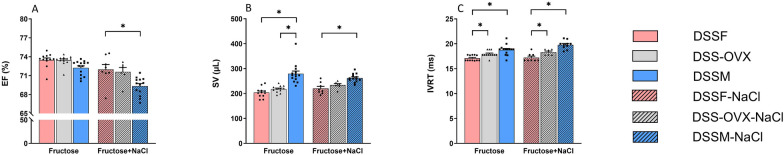
End-point measurements of left ventricular (LV) structural and functional parameters obtained from Dahl salt-sensitive (DSS) rats following diet intervention (8 weeks) using echocardiography from the parasternal short axis view in M-mode among the three sex groups. Ejection fraction **(**EF, **A)**, Stroke volume **(**SV, **B)**, Intraventricular relaxation time **(**IVRT, **C)**. Intact female (DSSF), ovariectomized female (DSS-OVX) and male (DSSM) rats in the control diet group were given standard chow (0.3% NaCl) and water supplemented with 10% fructose, while those in the high salt diet groups received a salt-supplemented diet (6% NaCl) along with fructose supplementation (DSSF-NaCl, DSS-OVX-NaCl and DSSM-NaCl). The values are shown as mean ± SEM, *n* = 6–14 in each group. *P < 0.05, 2-way ANOVA with Tukey's *post hoc* test.

Blood pressure measurements and echocardiography performed at baseline, before diet-interventions are presented in Table 2. As expected, there were structural and functional differences between female and male DSS rats at baseline (Table 2). At the study endpoint, LV mass, LV mass/tibia length, LVIDd, LVIDs, LVAWd, and LVPWd, LVESV, were increased with high-salt diet, whereas the slight increase in LVEDV was not significant (Figure 2 and Table 3). As a consequence, we observed a slight reduction in LV systolic function, measured by EF and FS, with high-salt diet, which, however, did not affect significantly pump performance (SV) in females but resulted in a reduction in males (with significant sex-diet interaction *p* = 0.04) (Figure 3 and Table 3). A slightly higher IVRT was also found under NaCl diet, suggesting prolonged LV relaxation (Figure 3 and Table 3). RWT tended to decrease in intact female rats with high-salt diet, whereas it increased in females with ovariectomy and substantially more in males, revealing a significant sex-diet interaction (*p* < 0.001) (Figure 2 and Table 3).

**Table 2:** Baseline heart rate, blood pressure (BP) and left ventricular (LV) structural and functional parameters assessed by transthoracic echocardiography obtained from Dahl salt-sensitive (DSS) female (DSSF) and male (DSSM) rats at age 9–10 weeks before any diet intervention. Data are presented as mean ± SEM.

Parameter	DSSF (*n* = 38–50)	DSSM (*n* = 27–29)
Heart rate (BPM)	411 ± 6	393 ± 6*
Systolic BP (mmHg)	141 ± 3	151 ± 3*
Diastolic BP (mmHg)	98 ± 2	103 ± 3
LVIDd (mm)	6.8 ± 0.1	7.7 ± 0.1*
LVESV (µL)	61 ± 1	92 ± 2*
LVEDV (µL)	238 ± 4	314 ± 6*
SV (µL)	177 ± 3	222 ± 4*
CO (mL/min)	72 ± 1	87 ± 2*
LV Mass (mg)	561 ± 9	832 ± 18*
LV mass/tibia length (mg/cm)	136 ± 2	177 ± 8*
LVPWd (mm)	1.35 ± 0.02	1.57 ± 0.03*
RWT	0.40 ± 0.01	0.40 ± 0.01
EF (%)	74.3 ± 0.2	70.7 ± 0.3*
FS (%)	44.3 ± 0.1	41.5 ± 0.2*
IVRT (ms)	14.3 ± 0.1	15.7 ± 0.2*

LV internal diameter in diastole (LVIDd), LV end-systolic volume (LVESV), LV end-diastolic volume (LVEDV), stroke volume (SV), LV mass was obtained from echocardiographic measurements, LV anterior wall in diastole (LVAWd), LV posterior wall in diastole (LVPWd), ejection fraction (EF), fractional shortening (FS), intraventricular relaxation time (IVRT). Significant difference *p < 0.05 vs. DSSF group.

**Table 3:** Endpoint left ventricular (LV) structural and functional parameters assessed by transthoracic echocardiography obtained from Dahl salt-sensitive (DSS) rats following diet intervention (8 weeks). Intact female (DSSF), ovariectomized female (DSS-OVX) and male (DSSM) rats in the control diet group were given standard chow (0.3% NaCl) and water supplemented with 10% fructose, while those in the high salt diet groups (+NaCl) received a salt-supplemented diet (6% NaCl) along with fructose supplementation. Data are presented as mean ± SEM, *n* = 6–14 in each group.

Parameter	Diet intervention	DSSF	DSS-OVX	DSSM	Diet *p* value
Heart rate (BPM)	Fructose only	393 ± 8	396 ± 6	402 ± 8	Sex n.s.
	Fructose + NaCl	415 ± 7	402 ± 15	411 ± 12	+NaCl n.s.
LVIDd (mm)	Fructose only	7.3 ± 0.1	7.5 ± 0.1	8.4 ± 0.1*^,#^	Sex *p* < 0.001
	Fructose + NaCl	7.6 ± 0.1	7.5 ± 0.1	8.3 ± 0.1*^,#^	+NaCl *p* = 0.04
LVIDs (mm)	Fructose only	4.08 ± 0.05	4.20 ± 0.04	4.78 ± 0.08*^,#^	Sex *p* < 0.001
	Fructose + NaCl	4.35 ± 0.11	4.50 ± 0.03	4.95 ± 0.07*^,#^	+NaCl *p* = 0.001
LVESV (µL)	Fructose only	74 ± 2	79 ± 2	107 ± 4*^,#^	Sex *p* < 0.001
	Fructose + NaCl	86 ± 5	93 ± 2	116 ± 4*^,#^	+NaCl *p* < 0.001
LVEDV (µL)	Fructose only	278 ± 8	297 ± 6	387 ± 15*^,#^	Sex *p* < 0.001
	Fructose + NaCl	307 ± 13	327 ± 6	379 ± 8*^,#^	+NaCl n.s.
SV (µL)	Fructose only	204 ± 6	218 ± 4	280 ± 11*^,#^	Sex *p* < 0.001
	Fructose + NaCl	221 ± 8	234 ± 6	262 ± 5*	+NaCl n.s.
					Interaction *p* = 0.04
CO (mL/min)	Fructose only	80 ± 2	86 ± 2	112 ± 5*^,#^	Sex *p* < 0.001
	Fructose + NaCl	92 ± 4	94 ± 4	108 ± 3*	+NaCl n.s.
LV mass (mg)	Fructose only	738 ± 16	899 ± 15*	1,136 ± 42*^,#^	Sex *p* < 0.001
	Fructose + NaCl	812 ± 33	1,076 ± 10*	1,287 ± 27*^,#^	+NaCl *p* < 0.001
LV mass/tibia length (mg/cm)	Fructose only	181 ± 04	219 ± 4*	250 ± 10*^,#^	Sex *p* < 0.001
	Fructose + NaCl	194 ± 07	262 ± 3*	285 ± 06*	+NaCl *p* < 0.001
LVPWd (mm)	Fructose only	1.59 ± 0.04	1.77 ± 0.03*	1.81 ± 0.05*	Sex *p* < 0.001
	Fructose + NaCl	1.50 ± 0.06	1.90 ± 0.01*	2.06 ± 0.04*	+NaCl *p* = 0.01
					Interaction *p* < 0.001
RWT	Fructose only	0.44 ± 0.01	0.47 ± 0.01	0.43 ± 0.01^#^	Sex *p* < 0.001
	Fructose + NaCl	0.40 ± 0.02	0.49 ± 0.01*	0.50 ± 0.01*	+NaCl n.s.
					Interaction *p* < 0.001
EF (%)	Fructose only	73.5 ± 0.3	73.4 ± 0.2	72.2 ± 0.3	Sex *p* < 0.001
	Fructose + NaCl	72.0 ± 0.8	71.6 ± 0.7	69.3 ± 0.4*^,#^	+NaCl *p* < 0.001
FS (%)	Fructose only	43.7 ± 0.3	43.8 ± 0.2	43.0 ± 0.2	Sex *p* < 0.001
	Fructose + NaCl	42.6. ± 0.7	42.3 ± 0.6	40.6 ± 0.3*^,#^	+NaCl *p* < 0.001
IVRT (ms)	Fructose only	17.1 ± 0.2	18.0 ± 0.2*	18.8 ± 0.3*^,#^	Sex *p* < 0.001
	Fructose + NaCl	17.2 ± 0.3	18.3 ± 0.2*	19.7 ± 0.3*^,#^	+NaCl *p* = 0.02

LV internal diameter in diastole (LVIDd) and systole (LVIDs), LV end-systolic volume (LVESV), LV end-diastolic volume (LVEDV), LV stroke volume (SV), cardiac output (CO), LV mass was obtained from echocardiographic measurements, LV anterior wall in diastole (LVAWd), LV posterior wall in diastole (LVPWd), relative wall thickness (RWT),), ejection fraction (EF), fractional shortening (FS), intraventricular relaxation rate (IVRT). Significant difference *p < 0.05 vs. the DSSF and **^#^***p* < 0.05 vs. the DSS-OVX from the corresponding diet group. Interaction between diet and sex is indicated with *p*-values in the table.

### 3.3 Gene expression regulating structure and function

**Figure 4:**
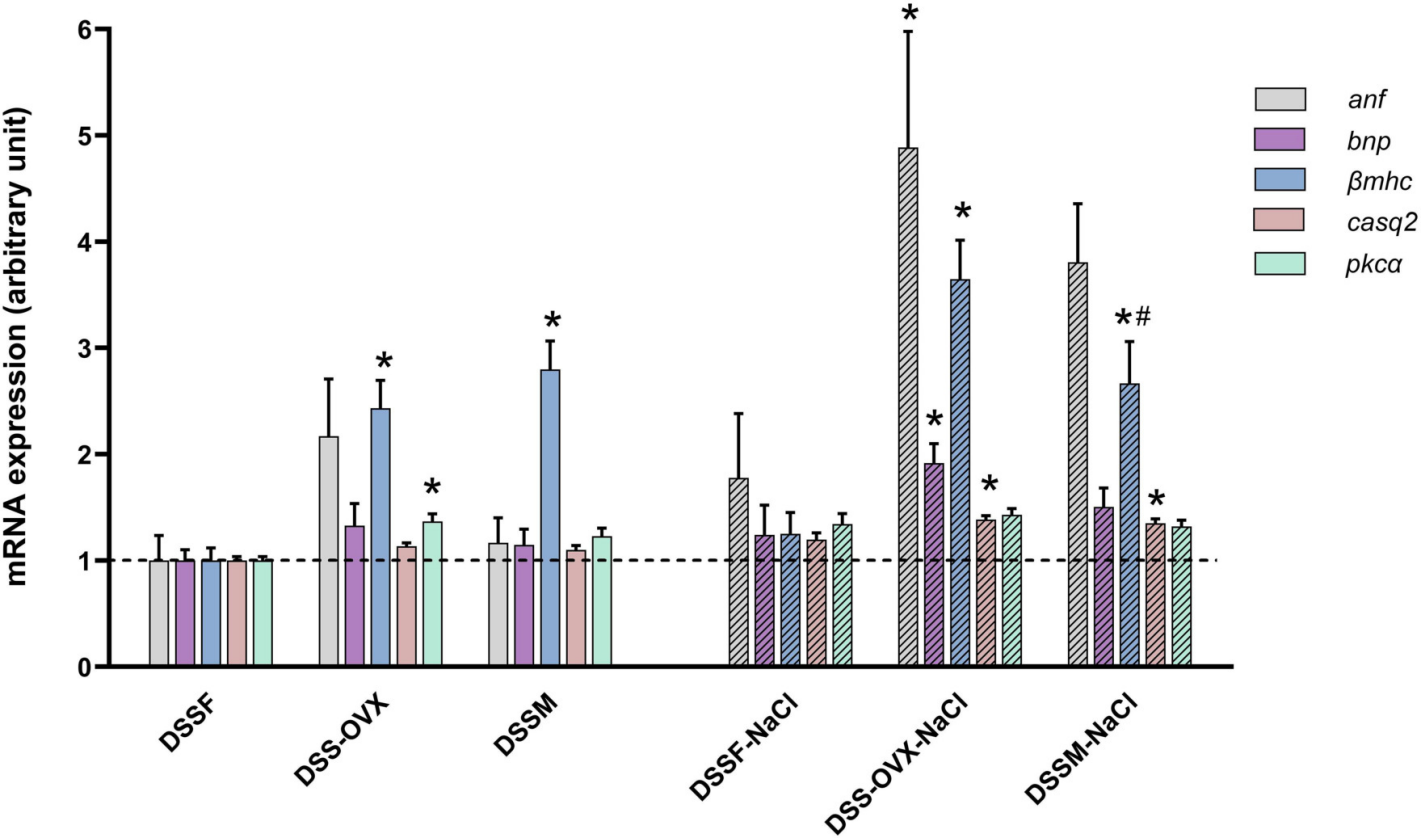
Gene expression related to heart function from the apical left ventricle of Dahl salt-sensitive (DSS) rats given a diet intervention (8 weeks). Expression is normalized to house-keeping genes and presented as change from the female group (DSSF, mean value adjusted to 1). Intact female (DSSF), ovariectomized female (DSS-OVX) and male (DSSM) rats in the control diet group were given standard chow (0.3% NaCl) and water supplemented with 10% fructose, while those in the high salt diet groups received a salt-supplemented diet (6% NaCl) along with fructose supplementation (DSSF-NaCl, DSS-OVX-NaCl and DSSM-NaCl). Calsequestrin 2 (*casq2*), protein kinase C alpha (*pkca)*, brain natriuretic peptide (*bnp),* beta myosin heavy chain *(βmhc*)*,* atrial natriuretic factor (*anf).* The values shown mean ± SEM, *n* = 14–15 in each group, *p < 0.05 vs. corresponding DSSF and **^#^***p* < 0.05 vs. the DSS-OVX from the corresponding diet group, 2-way ANOVA with Tukey's *post hoc* test.

Table 4A and Figure 4 show that mRNA expression of atrial natriuretic peptide (*anf*)*,* brain natriuretic peptide (*bnp*)*,* alpha myosin heavy chain (*αmhc*)*,* phospholamban (*pln*)*,* calsequestrin 2 (*casq*)*,* protein kinase C alpha (*pkcα*), connexin 43 (*cx43*) were significantly higher expressed in the presence of high-salt diet.

**Table 4A:** mRNA expression of genes related to heart function in the apical left ventricle obtained from Dahl salt-sensitive (DSS) rats following diet intervention (8 weeks). Intact female (DSSF), ovariectomized female (DSS-OVX) and male (DSSM) rats in the control diet group were given standard chow (0.3% NaCl), and water supplemented with 10% fructose, while those in the high salt diet groups (+NaCl) received a salt-supplemented diet (6% NaCl) along with fructose supplementation. Data are normalized to house-keeping genes as mean ± SEM, *n* = 14–15 in each group.

Gene	Diet intervention	DSSF	DSS-OVX	DSSM	Diet *p* value
*anf*	Fructose only	0.7 ± 0.2	1.6 ± 0.4	0.9 ± 0.2	Sex *p* = 0.003 + NaCl
	Fructose + NaCl	1.3 ± 0.5	3.6 ± 0.8*	2.8 ± 0.4	*p* < 0.001
*bnp*	Fructose only	2.4 ± 0.2	3.2 ± 0.5	2.7 ± 0.4	Sex *p* = 0.03 + NaCl
	Fructose + NaCl	2.9 ± 0.7	4.6 ± 0.4*	3.6 ± 0.4	*p* = 0.013
*αmhc*	Fructose only	119 ± 9	118 ± 6	100 ± 7	Sex n.s.
	Fructose + NaCl	142 ± 9	126 ± 6	127 ± 5	+NaCl *p* = 0.002
*βmhc*	Fructose only	2.3 ± 0.3	5.5 ± 0.6*	6.3 ± 0.6*	Sex *p* < 0.001
	Fructose + NaCl	2.8 ± 0.5	8.3 ± 0.8*	6.0 ± 0.9*^,#^	+NaCl n.s.
*pln*	Fructose only	17.8 ± 0.5	17.9 ± 0.5	16.2 ± 0.5	Sex n.s.
	Fructose + NaCl	20.3 ± 2.0	16.2 ± 0.7	15.9 ± 0.7	+NaCl *p* = 0.001
*casq2*	Fructose only	4.8 ± 0.2	5.4 ± 0.2	5.3 ± 0.2	Sex *p* < 0.001
	Fructose + NaCl	5.7 ± 0.3	6.6 ± 0.2*	6.4 ± 0.2*	+NaCl *p* < 0.001
*pkcα*	Fructose only	0.054 ± 0.002	0.074 ± 0.004*	0.066 ± 0.004	Sex *p* = 0.006 + NaCl
	Fructose + NaCl	0.073 ± 0.005	0.077 ± 0.003	0.071 ± 0.003	*p* = 0.005
*cx43*	Fructose only	1.9 ± 0.1	2.0 ± 0.1	1.7 ± 0.1	Sex n.s.
	Fructose + NaCl	2.1 ± 0.1	1.4 ± 0.1	1.5 ± 0.1	+NaCl *p* < 0.001

Atrial natriuretic peptide (*anf*)*,* brain natriuretic peptide (*bnp*)*,* alpha myosin heavy chain (*αmhc*)*,* beta myosin heavy chain (*βmhc*)*,* phospholamban (*pln*)*,* calsequestrin 2 (*casq2*)*,* protein kinase C alpha (*pkcα*), connexin 43 (*cx43*). Significant difference *p < 0.05 vs. the DSSF and **^#^***p* < 0.05 vs. the DSS-OVX from the corresponding diet group. Interaction between diet and sex is indicated with *p*-values in the table.

With respect to the three different sexual situations, DSS males and DSS-OVX exhibited increased expression of *βmhc* compared to intact DSSF with no directly detectable effect of diet. However, there was a borderline significant sex-diet interaction for *βmhc* expression (*p* = 0.053), sustained by the substantial increase in DSS-OVX and the decrease in DSSM.

### 3.4 Gene expression regulating fibrosis and inflammation

**Figure 5:**
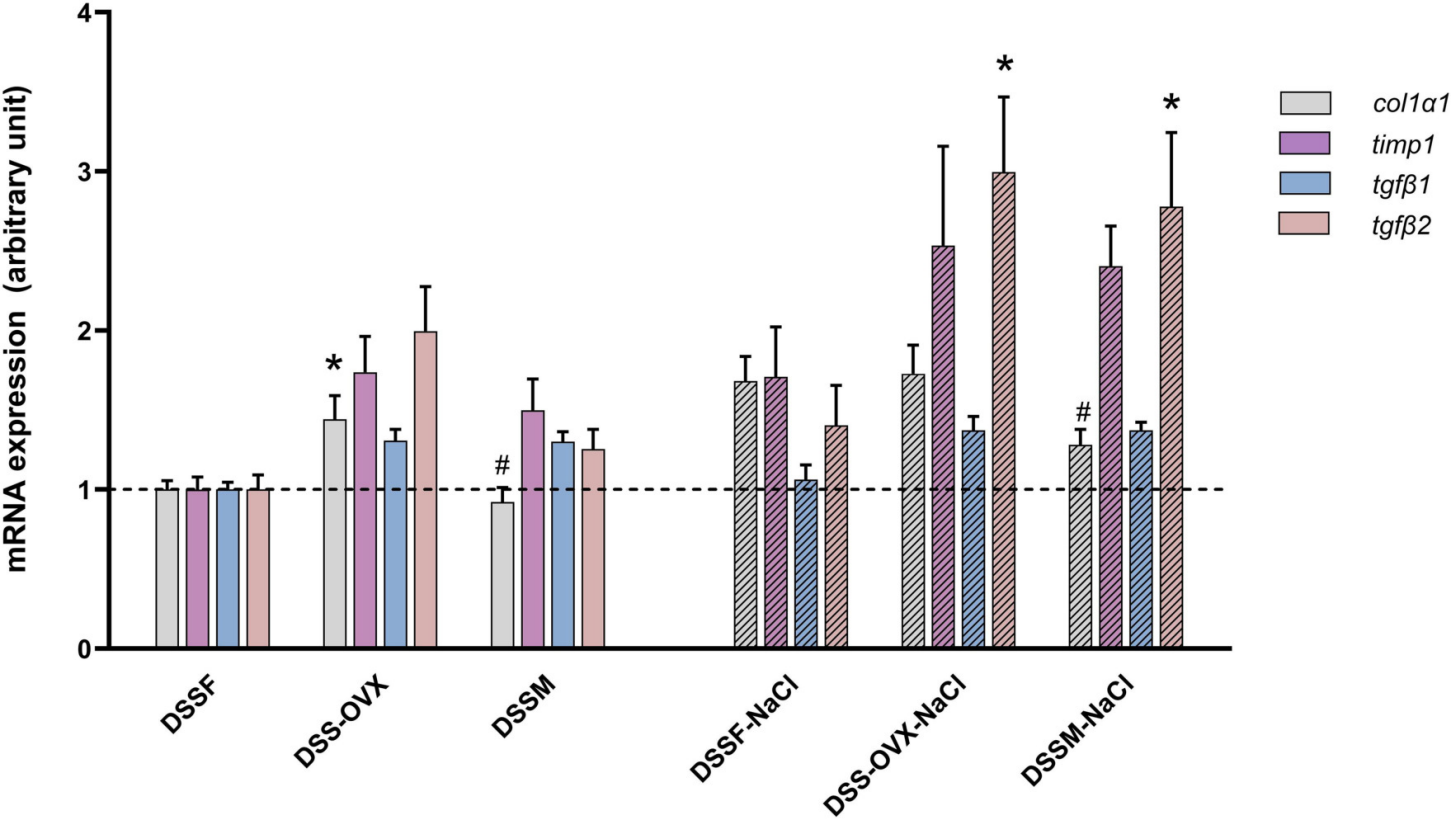
Gene expression related to fibrosis and inflammation from the apical left ventricle of Dahl salt-sensitive (DSS) rats given a diet intervention (8 weeks), expression is normalized to house-keeping genes and presented as change from the female group (DSSF, mean value adjusted to 1). Intact female (DSSF), ovariectomized female (DSS-OVX) and male (DSSM) rats in the control diet group were given standard chow (0.3% NaCl) and water supplemented with 10% fructose, while those in the high salt diet groups received a salt-supplemented diet (6% NaCl) along with fructose supplementation (DSSF-NaCl, DSS-OVX-NaCl and DSSM-NaCl). Collagen I, *α*1 (c*ol1α1*)*,* tissue Inhibitor of metalloproteinases 1 (*timp1)*, transforming growth factor *β1* and *β2 (tgf β1 and tgfβ2).* The values shown mean ± SEM, *n* = 14–15 in each group, *p < 0.05 vs. DSSF and **^#^***p* < 0.05 vs. the DSS-OVX from the corresponding diet group, 2-way ANOVA with Tukey's *post hoc* test.

Table 4B and Figure 5, show that high salt diet increase mRNA expression of collagen I (*col1*) and III (*col 3*)*,* tissue inhibitor of metalloproteinase1 (*timp1*), tumor necrosis factor *α* (*tnf α*), monocyte chemoattractant protein-1 (*mcp-1*) and transforming growth factor *β2 (tgfβ2*)*.* No significant difference could be detected for sex nor any significant sex-diet interaction.

**Table 4B:** mRNA expression of genes related to inflammation and fibrosis in the apical left ventricle obtained from Dahl salt-sensitive (DSS) rats following diet intervention (8 weeks). Intact female (DSSF), ovariectomized female (DSS-OVX) and male (DSSM) rats in the control diet group were given standard chow (0.3% NaCl), and water supplemented with 10% fructose, while those in the high salt diet groups (+NaCl) received a salt-supplemented diet (6% NaCl) along with fructose supplementation. Data are normalized to house-keeping genes as mean ± SEM, *n* = 14–15 in each group.

Gene	Diet intervention	DSSF	DSS-OVX	DSSM	Diet *p* value
*col1*	Fructose only	0.39 ± 0.02	0.57 ± 0.06*	0.39 ± 0.03^#^	Sex *p* = 0.001
	Fructose + NaCl	0.66 ± 0.06	0.68 ± 0.07	0.50 ± 0.04^#^	+NaCl *p* < 0.001
*col3*	Fructose only	0.25 ± 0.02	0.35 ± 0.04	0.35 ± 0.08	Sex n.s.
	Fructose + NaCl	0.43 ± 0.04	0.45 ± 0.05	0.37 ± 0.02	+NaCl *p* = 0.007
*fn-1*	Fructose only	0.024 ± 0.002	0.033 ± 0.004	0.032 ± 0.004	Sex n.s.
	Fructose + NaCl	0.033 ± 0.004	0.043 ± 0.008	0.034 ± 0.004	+NaCl n.s.
*timp1*	Fructose only	0.040 ± 0.003	0.070 ± 0.009	0.060 ± 0.008	Sex n.s.
	Fructose + NaCl	0.069 ± 0.013	0.102 ± 0.025	0.097 ± 0.010	+NaCl *p* = 0.004
*tnf* α	Fructose only	0.004 ± 0.000	0.005 ± 0.001	0.005 ± 0.001	Sex n.s.
	Fructose + NaCl	0.007 ± 0.001	0.006 ± 0.001	0.006 ± 0.000	+ NaCl *p* = 0.001
*mcp-1*	Fructose only	0.003 ± 0.000	0.005 ± 0.001	0.003 ± 0.000	Sex n.s.
	Fructose + NaCl	0.008 ± 0.003	0.008 ± 0.001	0.006 ± 0.001	+NaCl *p* = 0.003
*tgfβ1*	Fructose only	0.093 ± 0.004	0.121 ± 0.007*	0.120 ± 0.006*	Sex *p* < 0.001
	Fructose + NaCl	0.106 ± 0.004	0.127 ± 0.008*	0.127 ± 0.005*	+NaCl n.s.
*tgfβ2*	Fructose only	0.053 ± 0.005	0.106 ± 0.015	0.067 ± 0.007	Sex *p* < 0.001
	Fructose + NaCl	0.075 ± 0.013	0.159 ± 0.025*	0.148 ± 0.025*	+NaCl *p* < 0.001

Collagen I (*col1*) and III (*col 3*), fibronectin-1 (*fn-1*), tissue inhibitor of metalloproteinase 1 (*timp1*)*,* tumor necrosis factor α (*tnf α*)*,* monocyte chemoattractant protein-1 (*mcp-1*), transforming growth factor β1 (*tgfβ1*) and β2 *(tgfβ2*)*.* Significant difference *p < 0.05 vs. the DSSF and **^#^***p* < 0.05 vs. the DSS-OVX from the corresponding diet group.

### 3.5 Correlation between gene expression and myocardial geometry and function

**Figure 6:**
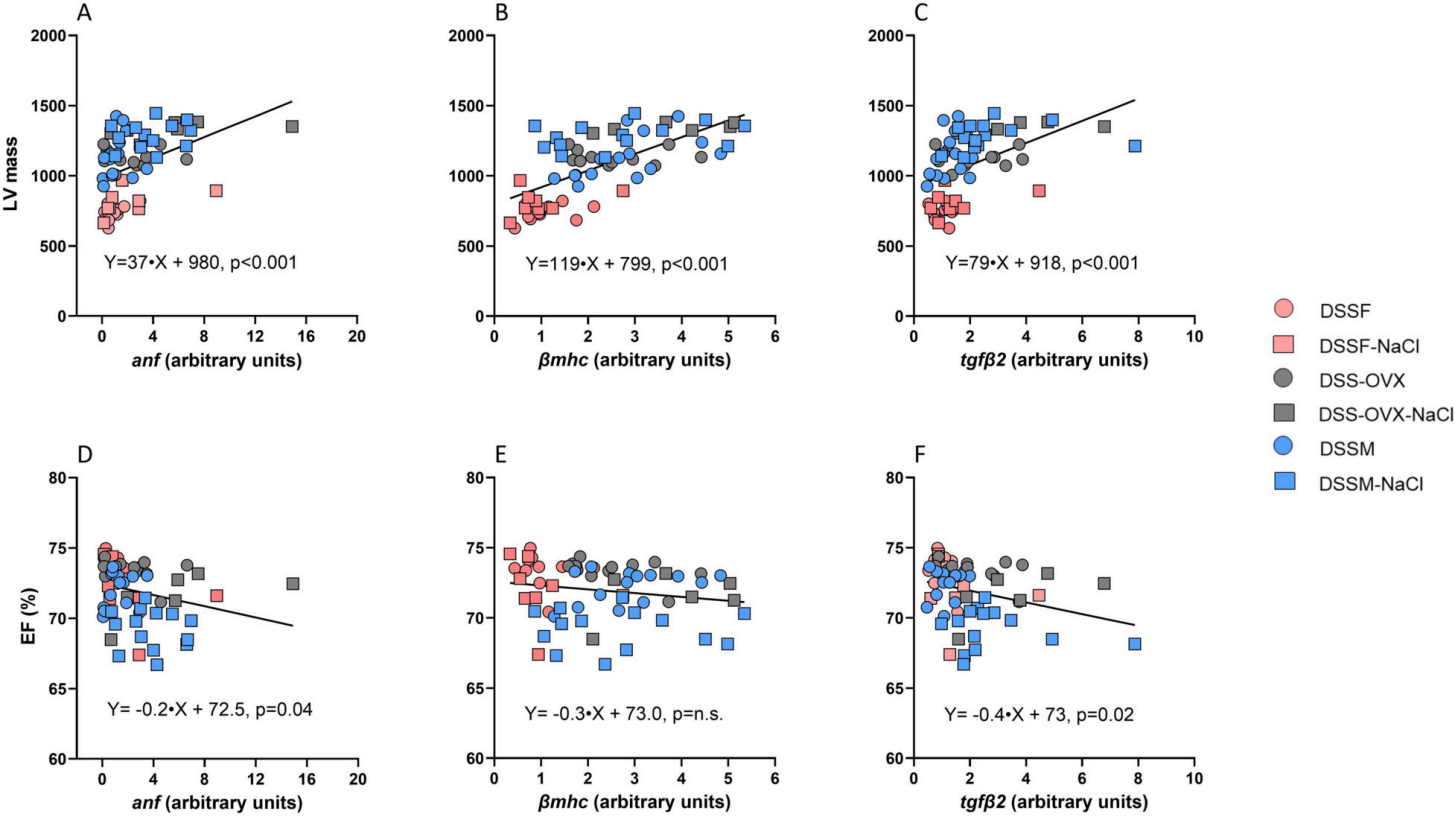
Simple linear correlations between echocardiographic measurements of left ventricular (LV) structure **(A–C)**, and function **(D–F)**, and myocardial mRNA expression of genes from the same individuals of Dahl salt-sensitive (DSS) rats given diet interventions (8 weeks). Intact female (DSSF), ovariectomized female (DSS-OVX) and male (DSSM) rats in the control group were given standard chow (0.3% NaCl) and water supplemented with 10% fructose, while those in the high salt diet groups received a salt-supplemented diet (6% NaCl) along with fructose supplementation (DSSF-NaCl, DSS-OVX-NaCl and DSSM-NaCl). LV mass (LV Mass), and ejection fraction (EF), obtained from echocardiographic measurements, mRNA expression of atrial natriuretic peptide (*anf*), *β*-myosin heavy chain (*βmhc*) and transforming growth factor *β2* (*tgfβ2*) are normalized to house-keeping genes and presented as change from the female group (DSSF, mean value adjusted to 1). Data are single values with regression-equations with corresponding *p*-values for all individuals in experimental groups.

Figure 6 includes individual data points from the hearts across all experimental groups with corresponding equations and *p*-values. We found LV mass to be positively correlated with myocardial *anf* (*p* < 0,001), *βmhc* (*p* < 0.001) and *tgfβ2* (*p* < 0.001) expression (Figure 6). EF was negatively correlated with expression of *anf* (*p* = 0.04) and t*gfβ2* (*p* = 0.02, Figure 6).

We also performed correlation analysis in subgroups of female, OVX and male DSS on either diets (Table 5). The correlation between *anf* and LV mass was also confirmed for females (*p* = 0.02), OVX (*p* = 0.03) and males (*p* = 0.01) on merged normal and high-salt diet. The correlation between LV mass and the expression of *βmhc* was substantially diluted in all groups, remaining significant only for OVX DSS rats (*p* = 0.02). The correlation between *tgfβ2* expression and LV mass also remained significant only in OVX DSS rats (*p* = 0.04) across diets. The correlation between EF and *anf* remained significantly in females (*p* = 0.05) and male (*p* = 0.01) DSS rats, whereas the correlation with EF and *tgfβ2* expression was significant only in male DSS rats (*p* = 0.01).

**Table 5:** Simple linear regression-equations with corresponding *p*-values from correlations between echocardiographic measurements of left ventricular (LV) mass and systolic function and myocardial mRNA expression of genes from the same individuals of Dahl salt-sensitive (DSS) rats given diet interventions (8 weeks). Intact female (DSSF), ovariectomized female (DSS-OVX) and male (DSSM) rats in the control group were given standard chow (0.3% NaCl) and water supplemented with 10% fructose, while those in the high salt diet groups received a salt-supplemented diet (6% NaCl) along with fructose supplementation (DSSF-NaCl, DSS-OVX-NaCl and DSSM-NaCl).

Variables	Groups		
	DSSF and DSSF-NaCl	DSS-OVX and DSS-OVX- NaCl	DSSM and DSSM-NaCl
*anf*-LV Mass	Y = 21•X + 734, *p* = 0.02	Y = 17•X + 1,139, *p* = 0.03	Y = 32•X + 1,130, *p* = 0.01
*βmhc*-LV Mass	Y = 39•X + 727, *p* = 0.20	Y = 55•X + 1,035, *p* = 0.02	Y = 38•X + 1,104, *p* = 0.09
*tgfβ_2_*-LV Mass	Y = 38•X + 721, *p* = 0.08	Y = 38•X + 1,096, *p* = 0.04	Y = 36•X + 1,136, *p* = 0.06
*anf*-EF	Y = −0.40•X + 73.52, *p* = 0.05	Y = −0.09•X + 73.40, *p* = 0.17	Y = −0.45•X + 71.89, *p* = 0.01
*tgfβ_2_*-EF	Y = −0.64•X + 73.66, *p* = 0.19	Y = −0.12•X + 73.15, *p* = 0.58	Y = −0.63•X + 72.01, *p* = 0.01

LV mass was obtained from echocardiographic measurements, ejection fraction (EF). mRNA expression of atrial natriuretic peptide (*anf*), β-myosin heavy chain (*βmhc*) and transforming growth factor β2 (*tgfβ2*) were normalized to house-keeping genes and presented as change from the female group (DSSF, mean value adjusted to 1).

## 4 Discussion

In this study, we report that cardiac remodelling response to diets differed between males and females, and in females it was also dependent on whether ovaries were intact or not. The results show that while blood pressure increases on high-salt diet in both male and female rats, intact ovaries dampen the myocardial response to a mild hypertensive stimulus alone, as well as to the marked hypertensive stimulus induced by high-salt diet whereas loss of ovaries increased the response significantly. Increasing the hypertensive stress stimulus by elevating salt in the diet led to concentric hypertrophic remodelling in males and OVX females, but not in ovary intact females. The difference between the groups in concentric remodelling and hypertrophy were not a direct function of marked differences in blood pressure at endpoint but were observed in parallel with a significant increase in transcription of several genes related to heart failure markers and inflammation such as *anf, bnp, βmhc, and tgfβ2.*

The role of sex and menopausal status in the development of salt-sensitive hypertension has lately received increasing interest in cardiovascular epidemiology (5, 26). Few previous experimental studies have studied the combined stress of fructose and high-salt diet on the pathophysiology of cardiac remodeling taking both sex and gonadal status into account. Xu et al., tested the effect of 12 weeks of elevated fructose-diet alone (60%) with normal salt intake and demonstrated a marked increase in blood pressure and kidney damage in young (6 weeks of age) male DSS rats, but not in salt-resistant Dahl rats, suggesting a hypertensive response to fructose in male DSS rats (18). Sharma et al., observed increased mortality after high-salt diet (6%) in male DSS rats given a high fructose containing diet (70%) as compared to a diet with complex carbohydrate content (27).

Our protocol included salt supplementation with a moderately high salt content (6% NaCl) in the diet compared to the diet previously used in many experimental studies on DSS-rats (10, 11, 28–32). Also, the fructose was added to the drinking water in a concentration partly comparable to soft drinks. To further enhance the translational relevance, we conducted OVX on DSS females at an adult age (9 weeks of age), when their organs, heart and cardiomyocytes were considered fully developed and well adapted to ovary hormones influence. The intention was to make a less severe pathophysiological model of the early-stage hypertensive LV hypertrophy, with a milder stress, especially for mimicking pre- and post-menopausal female hearts. It is well described in the literature that the age of experimental animals is one of the important factors in the pathogeneses of experimental hypertension and unfortunately studies in DSS rat is often performed on younger animals (33). The current relatively high-salt diet in our study was well tolerated; it did not have a significant negative effect on body weight development compared to fructose alone and we did not observe any mortality in the corresponding subgroups.

We did not find overt differences in MAP between DSS-OVX and DSSF rat with intact ovaries comparing fructose alone and addition of high-salt. This is in apparent contrast with our previous studies on DSS rats (11), and other studies investigating the effects of pressure and volume loads following OVX in rats (34, 35). It also differs from Sasaki et al., who reported a salt-independent rise in systolic blood pressure after OVX that was prevented by estrogen supplementation (30). These discrepancies can be attributed to the milder salt exposure in our study combined with the fructose diet, which is a common mild hypertensive stimulus for all groups, and difference in animal age, timing of OVX, hemodynamic endpoints and measurement modalities (systolic blood pressure vs. MAP). In line with this, Elkholey et al., reported comparable blood pressure in male and female DSS rats after 6 weeks of a high-salt (8% NaCl) diet, consistent with our findings showing similar MAP across male, intact, and OVX females (36). We did however find differences in both liver weight and kidney weight in our study following high-salt diet with fructose. Although kidney remodeling is well described following salt loading in DSS rats (37), structural remodeling of liver regarding the role of sex and gonadal status has not been well studied.

Baseline echocardiography values in 9–10-week-old DSS rats were consistent with previously published data from comparable studies (10, 38, 39). Despite the lack of marked differences in MAP, salt supplementation in the diet led to significant increases in LV weight at autopsy and echocardiography-based LV mass, confirming the presence of heart hypertrophy, indicative of structural cardiac remodeling (10, 40, 41). The DSS rat model develops a type of hypertension which is sustained by a combination of hemodynamic volume and pressure overload. The degree of influence of the two hemodynamic stressors are also depending on sex hormonal status and time of exposure. Sex differences in heart-remodeling have been tested using a variety of experimental models, but in most studies ovary function was maintained intact. In female Wistar rats, OVX combined with transverse aortic constriction, a model of near pure pressure overload, significantly reduced nitric oxide synthase protein kinase B (Akt) activity and LV function compared to ovary intact females (42), an effect thought to be due to reduced activity of intracellular *σ*_1_ receptors in ventricular cardiomyocytes. Vlachovsky et al., examined white blood cells and kidney function in female Wistar rats OVX at age 8–9 weeks and exposed shortly to increased salt at age 20–21 weeks (43). They conclude that OVX alone significantly impairs sodium handling proteins at the cellular level and reduces the ability of the kidney to secrete sodium compared to ovary intact females (43). This fits well with our previous protocol exposing OVX DSS rats to prolonged (16 weeks) control diet or high-salt diet and observing marked increase in SV and CO (11). In contrast, the present 8-week milder diet caused no significant SV and CO difference between OVX and ovary intact females.

Some of the earlier studies indicated that male sex was associated with increased LV susceptibility to hemodynamic overload compared to females (36, 44, 45), but the present study demonstrate that this seem influenced by presence or absence of ovary activity. Indeed, the increased susceptibility of women compared with males with hypertensive heart hypertrophy is confirmed clinically in the Campania Salute Network study which used major cardiovascular events as endpoint (46). Also, with adequate treatment of hypertension women tend to have more residual heart hypertrophy (47). The significant differences between females with intact ovaries on one side and males and OVX females on the other side were also present at endpoint under fructose stress alone, confirming the role of sex and hormonal influence in the development of mild LV hypertrophy in the present study. The observed endpoint differences between male and female DSS rats align with known sex-specific cardiovascular responses in experimental studies, where male DSS rats typically exhibit greater LV mass and chamber dimensions, while females-especially those with intact ovarian function show relative protection against hypertensive remodeling (6, 11, 35, 36).

Consistent with our observations, de Simone G et al., reported that male DSS rats fed 8% NaCl diet for 8 weeks developed a marked increase in LV mass (from 1.6 ± 0.6 to 2.1 ± 0.7 g/kg) (10). Levanovich et al., examined the combined effect of fructose and mildly high-salt diet (20% fructose and 4% Na^+^) in male Sprague-Dawley rats and demonstrated increased LV mass and wall thickness, consistent with hypertrophic remodeling and myocardial collagen accumulation (40). In that model, systolic function (EF and FS) was largely preserved, likely due to the prevalent concentric geometry, reducing myocardial afterload and preserving LV systolic function. In contrast, consistent with our findings, diastolic parameters indicated early diastolic dysfunction (40). Our results paralleling the above findings indicate that combination of metabolic challenge with moderately high-salt diet contribute to address cardiac response to hemodynamic overload toward concentric LV geometry and subtle diastolic impairment, especially in males, in line with prior reports (40).

In addition to the observed LV structural and functional modifications, the present study also revealed distinct gene expression patterns influenced by both combined diet and sex. In our previous study, we observed an increase in collagen mRNA expression and perivascular fibrosis in cardiac tissue following 16 weeks of high- (8%) salt diet (11). In line with the observed cardiac remodeling in the current study, we also found expressions of several genes known to be co-regulated with heart hypertrophy and pathology to be increased in the LV tissue following high-salt diet, despite its shorter duration and lower salt content-diet compared to our previous study (11).

The salt-diet together with fructose-supplementation led to the re-expression of fetal gene isoforms in the adult LV, as evidenced by the overexpression of *βmhc* and *tgfβ2* in our study. This phenomenon aligns with findings from other studies that have documented the re-expression of various fetal gene protein isoforms typically expressed in the embryonic heart during cardiac hypertrophy (48, 49). In addition, expression of inflammatory markers is also evident in our study and is known to be a marker of progression to heart failure (50). Interestingly, the highest effect of high-salt diet is found in the DSS-OVX rats that presents with a rate of expression more similar to DSSM than to DSSF, suggesting that estrogen deficiency increases susceptibility to LV remodeling under moderately high-salt intake. Especially *tgfβ2* was significantly upregulated in OVX female hearts compared to those of ovary-intact females. *Tgf-β* has been shown to drive the transformation of fibroblasts into myofibroblasts, playing a crucial role in the development of cardiac fibrosis and a crucial factor in LV-remodelling (51).

The mRNA expression of *mcp-1* was elevated in all salt treated animals as compared to corresponding groups without salt, suggesting migration of pro-inflammatory cells into myocardium, a key factor in heart failure progression (52). A study in a hypertensive rat model demonstrated increased expression of proinflammatory cytokine genes, including *mcp-1* and osteopontin, in the hearts of DSS rats, accompanied by macrophage infiltration into the perivascular spaces of coronary vessels (41). Macrophages contribute to the production of cytokines such as *tgf-β1* and interleukin- IL1*β* which are also involved in myocyte hypertrophy (53). Several other genes, such as *timp-1*, were also significantly upregulated under high-salt diet, particularly in the DSS-OVX and DSSM, indicating dysregulation in the extracellular matrix degradation and collagen deposition in perivascular spaces (48, 49, 51, 54).

Furthermore, *anf* expression was 2.8-fold higher in the DSS-OVX on high-salt diet as compared to the DSSF, and even higher than in DSSM. Sangaralingham et al., showed increased hypertrophic response and increased expression in the natriuretic peptide system in the ventricles in pro-ANF heterozygote male and OVX female mice exposed to high-salt but no response in estradiol treated OVX female mice, indicating complex relationship between estradiol, sodium, and the natriuretic peptide system of the ventricles (55).

### 4.1 Limitation of the study

One of the limitations of the present study is that food and water intake were not measured, which could influence variations in blood pressure due to differences in salt consumption, changes in appetite and physical activity over time. Despite these potential variations, by the end of the study, all animals within each diet group displayed comparable MAP level. Notably, there were no clear cases of hypertensive heart failure. Age-matched control groups without dietary modification were not included. Therefore, we cannot distinguish the dietary effect of fructose from the age-dependent gradual increase in cardiovascular stress in Dahl rats (34). For normalization or indexing, we have used tibia length, but we have also tested the use of a validated formula for surface area of the rat based on body weight and obtained comparable results (23). In the present study, we examined gene expression of selected genes in hypertension induced heart hypertrophy based on the literature and our previous studies, and the selection might be subject to bias. Also, while a change in mRNA expression indicates alteration in upstream signaling, transcription factors and the gene promotor, downstream changes at the corresponding protein level were not tested in our study. Nonetheless, the heart function data fits well with the observed changes in gene expression. Some studies suggest that females are potentially more susceptible to cardiac impacts and males more affected by renal impacts, but in the present study, we did not test kidney function (56, 57). Finally, we did not substitute the OVX rats with hormone and can only speculate about the role of estrogen when explaining the findings.

## 5 Conclusion

This study demonstrates that exposure to a combined fructose and high-salt diet induces early cardiac remodeling in DSS rats, with marked sex- and gonadal-status dependent differences. While intact females exhibited relative resistance to concentric hypertrophic remodeling and a less pronounced activation of hypertrophy- and fibrosis-associated gene expression compared with OVX females and males. OVX females showed significantly higher structural remodeling and molecular activation, often resembling the phenotype observed in males. These differences were present despite comparable blood pressure at endpoint, suggesting that loss of ovarian hormones makes the heart more vulnerable to hypertensive and metabolic stress beyond what can be explained by blood pressure alone. The distinct cardiac responses observed in OVX females compared with intact females, and their close resemblance to the male phenotype, further underscore the critical role of ovarian hormones in modulating cardiac remodeling under hemodynamic and metabolic stress.

## Data Availability

The raw data supporting the conclusion of this article are available from the corresponding author upon reasonable request.
