# Supplementary material for: Fructose and salt induce sex- and ovary dependent cardiac hypertrophy in Dahl salt-sensitive rats
Source: Front Cardiovasc Med. 2026 Mar 18;13:1753554. doi: 10.3389/fcvm.2026.1753554 (PMC13039016; doi:10.3389/fcvm.2026.1753554)
Supplement: Supplementary file 1 [file Table1.docx]

| **Supplementary Table 1.** Primers used for rt-PCR of genes in left ventricular biopsies | | |
| --- | --- | --- |
| Gene abbreviation | Primer | Corresponding protein |
| COL1A1 | \| CATGTTCAGCTTTGTGGACCT \| \| --- \| \| GCAGCTGACTTCAGGGATGT \| | Collagen I, α1 |
| COL3A1 | \| TCCCCTGGAATCTGTGAATC \| \| --- \| \| TGAGTCGAATTGGGGAGAAT \| | Collagen III, α1 |
| FN1 | \| CAGCCCCTGATTGGAGTC \| \| --- \| \| TGGGTGACACCTGAGTGAAC \| | Fibronectin 1 |
| TIMP-1 | \| CAGCAAAAGGCCTTCGTAAA \| \| --- \| \| TGGCTGAACAGGGAAACACT \| | Tissue inhibitor of metalloproteinase 1 |
| TGFB1 | \| AAGAAGTCACCCGCGTGCTA \| \| --- \| \| TGTGTGATGTCTTTGGTTTTGTCA \| | Transforming growth factor β1 |
| TGFB2 | \| ATCGATGGCACCTCCACATATG \| \| --- \| \| GCGAAGGCAGCAATTATCCTG \| | Transforming growth factor β2 |
| MCP1 | \| AGCATCCACGTGCTGTCTC \| \| --- \| \| GATCATCTTGCCAGTGAATGAGT \| | Monocyte chemoattractant protein-1 (MCP-1) |
| TNFα | \| GCCCAGACCCTCACACTC \| \| --- \| \| CCACTCCAGCTGCTCCTCT \| | Tumor necrosis factor α (adipokine and cytokine, alternative name cachectin), |
| NPPA | \| CAACACAGATCTGATGGATTTCA \| \| --- \| \| CGCTTCATCGGTCTGCTC \| | Natriuretric peptide A |
| CX43 | \| TTCATTGGGGGAAAGGCGTGAGGA \| \| --- \| \| GAAGCCGGCGCGCCAAAGTT \| | connexin 43 |
| NPPB | \| TGATTCTGCTCCTGCTTTTC \| \| --- \| \| GTGGATTGTTCTGGAGACTG \| | Natriuretric peptide B |
| GJA 1 | \| TTCATTGGGGGAAAGGCGTGAGGA \| \| --- \| \| GAAGCCGGCGCGCCAAAGTT \| | Gap junction protein, alpha 1 |
| PKCα | \| CAAGCAGTGCGTGATCAATGT \| \| --- \| \| GGTGACGTGCAGCTTTTCATC \| | Protein kinase C, alpha |
| CASQ2 | \| AGCCGCAGGACCAAGGAGGT \| \| --- \| \| AAGCCCCACCACGAGCAGGT \| | Calsequestrin 2 |
| PLN | \| AGCTCCCAGACTTCACACAAC \| \| --- \| \| CGCGAGCTGGCTCCTTTTAG \| | Phospholamban |
| MYH6 | \| CAAGGCAAACCTGGAGAAAG \| \| --- \| \| GGGTATAGGAGAGCTTGCCC \| | Myosin heavy chain 6 |
| MYH7 | \| GAGGAGAGGGCGGACATT \| \| --- \| \| ACTCTTCATTCAGGCCCTTG \| | Myosin heavy chain 7 |
